# Neuroprotective therapies in the NICU in preterm infants: present and future (Neonatal Neurocritical Care Series)

**DOI:** 10.1038/s41390-023-02895-6

**Published:** 2023-12-19

**Authors:** Eleanor J. Molloy, Mohamed El-Dib, Janet Soul, Sandra Juul, Alistair J. Gunn, Manon Bender, Fernando Gonzalez, Cynthia Bearer, Yvonne Wu, Nicola J. Robertson, Mike Cotton, Aoife Branagan, Tim Hurley, Sidhartha Tan, Abbot Laptook, Topun Austin, Khorshid Mohammad, Elizabeth Rogers, Karen Luyt, Pia Wintermark, Sonia Lomeli Bonifacio, on behalf of the Newborn Brain Society Guidelines and Publications Committee, Sonia Lomeli Bonifacio, Pia Wintermark, Hany Aly, Vann Chau, Hannah Glass, Monica Lemmon, Courtney Wusthoff, Gabrielle deVeber, Andrea Pardo, Melisa Carrasco, James Boardman, Dawn Gano, Eric Peeples

**Affiliations:** 1 Paediatrics, Trinity College Dublin, Trinity Research in Childhood Centre (TRICC), Dublin, Ireland; 2 Children’s Hospital Ireland (CHI) at Tallaght, Dublin, Ireland; 3 Neonatology, CHI at Crumlin, Dublin, Ireland; 4 Neonatology, Coombe Women’s and Infants University Hospital, Dublin, Ireland; 5 Department of Pediatrics, Brigham and Women’s Hospital, Harvard Medical School, Boston, MA USA; 6 Department of Neurology, Boston Children’s Hospital, Harvard Medical School, Boston, MA USA; 7 Department of Pediatrics, University of Washington, Seattle, WA USA; 8 Departments of Physiology and Paediatrics, School of Medical Sciences, University of Auckland, Private Bag 92019, Auckland, New Zealand; 9 Department of Neonatology, Wilhelmina Children’s Hospital, University Medical Center Utrecht, Utrecht University, Utrecht, The Netherlands; 10 Department of Neurology, Division of Child Neurology, University of California, San Francisco, California USA; 11 Division of Neonatology, Department of Pediatrics, Rainbow Babies & Children’s Hospital, Cleveland, Ohio USA; 12 Case Western Reserve University School of Medicine, Cleveland, Ohio USA; 13 Department of Neurology, University of California San Francisco, San Francisco, California USA; 14 Institute for Women’s Health, University College London, London, UK; 15 Centre for Clinical Brain Sciences, University of Edinburgh, Edinburgh, UK; 16 Department of Pediatrics, Duke University, Durham, North Carolina USA; 17 Wayne State University School of Medicine, Detroit, Michigan USA; 18 Department of Pediatrics, Women and Infants Hospital, Brown University, Providence, Rhode Island USA; 19 Department of Paediatrics, University of Cambridge, Cambridge, UK; 20 Section of Neonatology, Department of Pediatrics, University of Calgary, Calgary, Alberta Canada; 21 Department of Pediatrics, University of California, San Francisco Benioff Children’s Hospital, San Francisco, California USA; 22 Translational Health Sciences, University of Bristol, Bristol, UK; 23 Neonatology, University Hospitals Bristol and Weston NHS Foundation Trust, Bristol, UK; 24 Division of Neonatology, Montreal Children’s Hospital, Montreal, Quebec Canada; 25 McGill University Health Centre - Research Institute, Montreal, Quebec Canada; 26 Division of Neonatal and Developmental Medicine, Department of Pediatrics, Stanford University School of Medicine, Stanford, California USA; 27 Neonatology, Pediatrics, Stanford University School of Medicine, Palo Alto, CA USA; 28 Neonatology, Pediatrics/Newborn Medicine, Montreal Children’s Hospital, McGill University, Montreal, QC Canada; 29 Neonatology, Cleveland Clinic Children’s Hospital, Bratenahl, OH USA; 30 Neurology, Pediatrics, The Hospital for Sick Children, Toronto, ON Canada; 31 Neurology, Pediatrics, Epidemiology & Biostatistics, University of California, San Francisco, CA USA; 32 Neurology, Pediatrics, Population Health Sciences, Duke University School of Medicine, Durham, NC USA; 33 Neurology, Child Neurology, Stanford, Stanford, CA USA; 34 Neurology, Hospital for Sick Children, Toronto, ON Canada; 35 Neurology, Pediatrics, Ann & Robert H. Lurie Children’s Hospital of Chicago, Chicago, IL USA; 36 McCaul Neurology, University of Wisconsin, Madison, WI USA; 37 MRC Centre for Reproductive Health, Queen’s Medical Research Institute, University of Edinburgh, Edinburgh, UK; 38 Departments of Neurology and Pediatrics, University of California, San Francisco, USA; 39 Department of Pediatrics, University of Nebraska Medical Center, Omaha, NE USA

## Abstract

**Abstract:**

The survival of preterm infants has steadily improved thanks to advances in perinatal and neonatal intensive clinical care. The focus is now on finding ways to improve morbidities, especially neurological outcomes. Although antenatal steroids and magnesium for preterm infants have become routine therapies, studies have mainly demonstrated short-term benefits for antenatal steroid therapy but limited evidence for impact on long-term neurodevelopmental outcomes. Further advances in neuroprotective and neurorestorative therapies, improved neuromonitoring modalities to optimize recruitment in trials, and improved biomarkers to assess the response to treatment are essential. Among the most promising agents, multipotential stem cells, immunomodulation, and anti-inflammatory therapies can improve neural outcomes in preclinical studies and are the subject of considerable ongoing research. In the meantime, bundles of care protecting and nurturing the brain in the neonatal intensive care unit and beyond should be widely implemented in an effort to limit injury and promote neuroplasticity.

**Impact:**

- With improved survival of preterm infants due to improved antenatal and neonatal care, our focus must now be to improve long-term neurological and neurodevelopmental outcomes.
- This review details the multifactorial pathogenesis of preterm brain injury and neuroprotective strategies in use at present, including antenatal care, seizure management and non-pharmacological NICU care.
- We discuss treatment strategies that are being evaluated as potential interventions to improve the neurodevelopmental outcomes of infants born prematurely.

## Introduction

The cost and deleterious impact of preterm brain injury on individuals, their extended families and society cannot be overstated. Preterm neonates are among our most vulnerable citizens. Brain injury is far more common in preterm infants than at term and is associated with a greater risk of adverse outcomes.^1^ Preterm birth is one of the leading indicators of the health of a nation, as it is associated with high mortality and significant risks of brain injury and impaired brain development.^2–6^ In turn, this is associated with poor academic achievements and behavioral and mental health issues, all of which affect the quality of life and reduce earning capacity throughout life.^5,7–9^

**Fig. 1:**
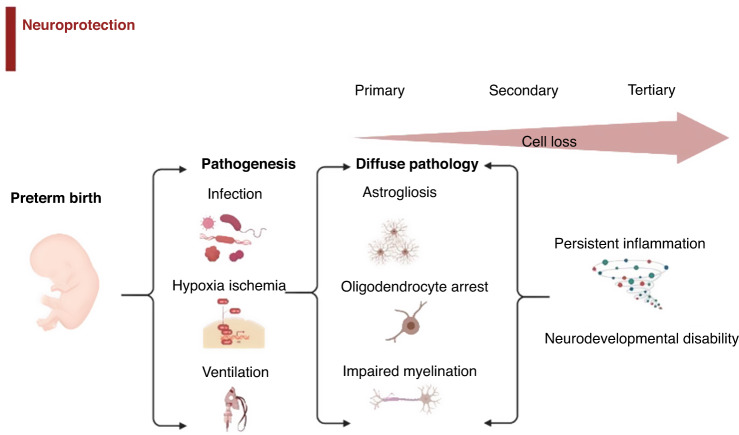
Brain injury in the premature infant can be caused by a variety of factors including infection and hypoxic injury. This can lead to diffuse damage, including cell loss via astrogliosis, oligodendrocyte arrest and impaired myelination. Intervention at each stage of injury and cell loss aims to prevent neurodevelopmental complications.

There is now considerable evidence that the pathogenesis of preterm brain damage is multifactorial, involving hypoxia-ischemia (HI), exposure to perinatal infection/inflammation, ventilation, and other technologies to support immaturity, and other perinatal events (Fig. 1).^10–14^ For example, in preterm infants, a 5-min Apgar score <7 is associated with a greater risk of death or cerebral palsy (CP) than a score ≥9. Equally, the extent and duration of inflammatory markers both at the time of birth and after are highly associated with severe neurodevelopmental disability.^15^

In modern cohorts, the most common underlying pathology is diffuse white matter injury (WMI), with astrogliosis, maturational arrest of oligodendrocytes and impaired myelination (Fig. 1).^16–18^ For example, in a large cohort of infants born before 30 weeks of gestation and/or less than 1500 g at birth, neurodevelopmental impairment at 7 years of age was associated with smaller total brain, cortical gray matter and white matter volumes on magnetic resonance imaging (MRI) at 7 years of age (*n* = 62, *p* < 0.05), and with reduced fractional anisotropy on diffusion-tensor imaging.^19^ The key mechanisms that underlie white matter dysmaturation in this context involve persistent neuroinflammation and loss of cellular trophic support.^20–22^

Although the incidence of CP has fallen in some populations of premature infants, absolute rates remain high, and overall neurodevelopmental outcomes have not clearly improved despite improvements in perinatal and neonatal clinical care.^23–25^ Structurally, CP is associated with diffuse white matter injury (WMI) and, in most severe cases, cystic WMI, previously called cystic periventricular leukomalacia.^26–28^ Experimentally, extensive cell loss most often occurred during the first few days after an injury, concomitant with secondary loss of cerebral oxidative metabolism.^29^ However, strikingly, in some studies, injury continued to evolve over days and weeks in a tertiary phase of injury.^20,30^ Consistent with this, clinically, cystic WMI can be seen on ultrasonography at a median of ~4 weeks after birth/injury, albeit more severe cysts tended to appear earlier.^31,32^ Non-cystic, diffuse WMI is most likely to be detected on MRI, although it can sometimes be identified on ultrasound. This apparent delay supports the hypothesis that both dysmaturation and, in some cases, severe injury may evolve over many weeks in the tertiary phase, potentially offering a very wide window of opportunity for intervention.^33^ Evidence gained from preclinical studies must be examined with precautions—while animals typically undergo a defined insult and then likely be sacrificed, injuries suffered by the human preterm infant are likely to be additive, with the risk for multiple, sequential insults in the same patient.

**Fig. 2:**
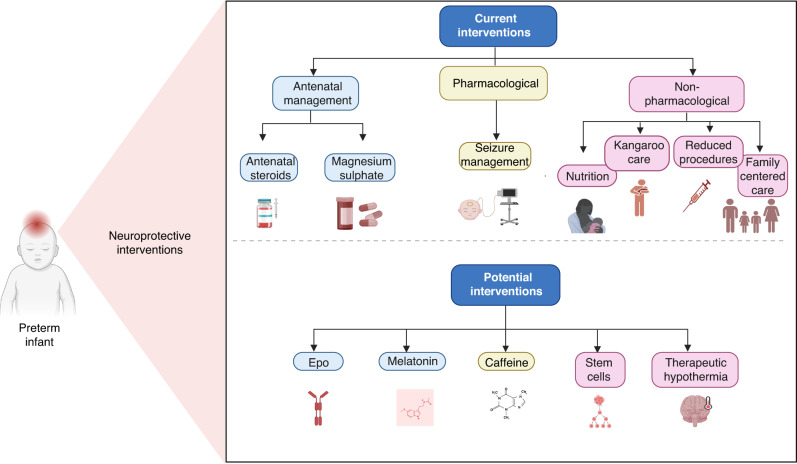
Current and potential therapies to prevent and treat brain injury in the preterm infant.

Interventions to protect the preterm brain with an aim to decrease long-term neurodevelopmental impairment can be divided into antenatal strategies, such as antenatal steroid use and magnesium sulfate, and postnatal treatment strategies, including those in routine use and potential agents for future use (Fig. 2).

## Antenatal management

### Antenatal steroids

The use of antenatal corticosteroids has strong short-term benefits and most likely improves the neurodevelopmental outcomes of preterm neonates. The primary aim is to accelerate fetal lung development but also, importantly, to stabilize the fetal neurovasculature.^34,35^ At present, a single course of antenatal steroids is recommended for all women at risk of delivering a premature infant by guidelines from all the major perinatal organizations, although there are minor differences in the recommendations for the upper and lower gestational age cutoffs.^36–39^ The Royal College of Obstetricians and Gynecologists (RCOG) suggests steroids in at-risk deliveries between 24+0 and 34+6 gestational ages. The American College of Obstetrics and Gynecology (ACOG) recommends steroids for pregnancies between 24+0 and 33+6 weeks gestation. They also recommend consideration to be given to providing steroids between 22+0 and 24+0 in consultation with the family and taking into consideration the family’s opinion on neonatal resuscitation. The World Health Organization (WHO) guidelines consider the setting of the pregnancy alongside gestational age, suggesting steroids are given between 24 and 34 weeks gestational age providing adequate care is available to the neonates. They acknowledge the potential for substantial clinical benefit in infants under 24 weeks and again advocate for shared decision-making between families and clinicians. In addition, recommendations vary for rescue dose after the initial course; the RCOG suggests considering it with caution only if the initial course was given at less than 26 weeks of gestation.^36^

A recent meta-analysis confirms that treatment with antenatal corticosteroids reduces the risk of perinatal death (risk ratio (RR) 0.85, 95% confidence interval (CI) 0.77 to 0.93) and the risk of neonatal death and respiratory distress syndrome (RR 0.71, 95% CI 0.65 to 0.78). They also probably reduce the risk of intraventricular hemorrhage (IVH) (RR 0.58, 95% CI 0.45 to 0.75) and developmental delay in childhood (RR 0.51, 95% CI 0.27 to 0.97).^40^

By contrast, their use in moderate to late premature infants is highly controversial, as the potential for benefit is much lower, and so risks may outweigh the benefits. The Antenatal Late Preterm Steroids (ALPS) trial, a randomized trial of 2827 infants between 34 and 36+5 weeks gestation, showed a decrease in respiratory morbidity in steroid-treated late premature infants, alongside an increase in episodes of hypoglycemia.^41^ A Finnish cohort study of over 600,000 children showed a higher risk of mental health and behavioral disorders in steroid-exposed infants, including autism spectrum disorder and attention-deficit/hyperactivity disorder.^42,43^ While the findings of this study have been debated, they point to the importance of avoiding therapeutic drift, especially at later gestations, when benefits will be less pronounced.

Postnatally, steroids are primarily used to decrease respiratory morbidity in premature infants with prolonged and high ventilatory requirements or oxygen use.^44,45^ Their use has remained controversial due to an apparent increase in the risk of longer-term neurodevelopmental impairments.^46^ Dexamethasone tends to be the first-line postnatal steroid for the prevention of bronchopulmonary dysplasia in the second week of life, mainly based on the findings of the DART trial.^47,48^ While underpowered for their outcomes, the DART trial showed improvements in respiratory status but no statistical difference in long-term outcomes. Thus, in practice, the risk of neurodevelopmental impairments with steroid use must be balanced against the improvement in short-term respiratory status. The challenge for neonatologists is in identifying the infants at the highest risk of poor neurodevelopmental outcomes secondary to poor respiratory status, in whom the benefits of steroid use likely outweigh the risks.

Finally, it is important to consider preclinical evidence that antenatal corticosteroids could have adverse effects for some premature infants exposed to acute HI. For example, maternal dexamethasone given 15 min after asphyxia in fetal sheep was associated with more severe damage in the hippocampus and basal ganglia and exacerbated loss of oligodendrocytes.^49^ Moreover, a single intramuscular injection of maternal dexamethasone 4 h before umbilical cord occlusion in preterm fetal sheep was associated with cystic brain injury, with increased numbers of seizures, worse brain activity and increased arterial glucose levels compared to preterm fetal sheep treated with vehicle before occlusion.^50^ These findings could be replicated by glucose infusions before asphyxia, suggesting that dexamethasone-induced hyperglycemia may transform diffuse injury into cystic brain injury after asphyxia, at least in the preterm fetal sheep. Unfortunately, acute asphyxia in a fetus after administration of antenatal steroids is typically unanticipated and unpreventable.

### Magnesium sulfate (MgSO_4_)

Antenatal magnesium sulfate (MgSO_4_) given prior to preterm birth for fetal neuroprotection is widely used, based on meta-analyses of trials of pre-eclampsia treatment or fetal neuroprotection involving 6131 neonates, including a recent individual participant meta-analysis (IPD-MA). Use of MgSO_4_ was associated with reduced risk of CP (RR 0.68, 95% confidence interval (CI) 0.54 to 0.87, 4601 neonates, 5 trials, number needed to benefit of 46).^51,52^ However, it is important to appreciate that, overall, MgSO_4_ did not significantly improve the risk of the composite outcome of death or CP (RR 0.94, 95% CI 0.85 to 1.05). Exclusion of a trial of treatment of maternal pre-eclampsia suggested, in the remaining four trials, a reduction in CP (RR 0.68, 95% CI 0.53 to 0.87) and in the combined risk of death or CP (RR 0.86, 95% CI 0.75 to 0.99). The findings from the most recent meta-analysis (5917 women giving birth to 6037 infants), including trial sequential analysis (TSA) of all RCTs of neuroprotective and other intent, provided additional evidence that antenatal MgSO_4_ in women at imminent risk for preterm delivery was associated with decreased risk of CP,^53^ but again the risk of death or CP was not significantly different (17.1% versus 18.3%, RR 0.88, 95% CI 0.75–1.02). There was no apparent benefit for short-term outcomes, such as severe intraventricular hemorrhage or periventricular leukomalacia.^52^ The specific causes of infant death in these studies are unknown, but could include redirection to comfort care in agreement between family and caregivers.

Overall, MgSO_4_ appears to be safe. There was no increase in the primary outcome of severe adverse maternal events related to treatment (including death or respiratory or cardiac arrest in 2 studies that reported this in a total of 1635 women). However, smaller studies suggest possible adverse intestinal effects in neonates that should be investigated carefully. For example, there was an apparent increase in spontaneous intestinal perforation in neonates born <26 weeks after the introduction of MgSO_4_.^54^ Further, in a subset of neonates <26 weeks from the BEAM trial, antenatal MgSO_4_ was associated with death and severe NEC.^55^ Moreover, the Korean neonatal research network reported that antenatal MgSO_4_ exposure was associated with meconium-related ileus in infants <25 weeks gestation.^56^ A recent meta-analysis of 38 non-randomized studies and six randomized trials involving 51,466 preterm infants did not, however, show an increase in gastro-intestinal pathology.^57^

Even if we consider the reduction in risk of CP to be the true effect of the intervention, there are concerns that this effect is small,^58^ with a risk reduction of all grades of CP from 6.7% to 4.7% (absolute reduction 2%, relative reduction 30%).^52^ The effect size for CP reduction is larger in extremely preterm infants, with an NNT of 31 below 28 weeks gestation. Recently, in a trial of treatment for preterm delivery at 30 to 34 gestation, MgSO_4_ was associated with a trend toward worse outcomes, with adjusted RR for death or disability of 1.19 [95% CI, 0.65 to 2.18],^59^ suggesting that we should consider limiting the use of MgSO_4_ to the highest risk gestations. Moreover, there was no improvement in school-age outcomes in magnesium sulfate-treated cohorts, albeit these studies were not powered to detect small effects and were challenged by loss to follow-up that might bias results.^60,61^

Importantly, there is a notable lack of consistent in vivo evidence demonstrating neuroprotection. A systematic review of animal studies found that the effect of MgSO_4_ treatment before or shortly after acute HI at term-equivalent gestation was inconsistent between studies.^62^ In rodent studies showing benefits, the majority were of extremely preterm gestation and used MgSO_4_ before the inflammatory/HI insult and did not report controlling temperatures. Given that magnesium is a potent vasodilator, this raises the possibility that it could induce neuroprotective hypothermia. MgSO_4_ given pre-insult, using a clinically relevant higher dose, induced strong preconditioning of the immature rat brain, which provided resistance to HI- and excitotoxicity-mediated injury.^63^ A small incremental benefit of MgSO_4_ + hypothermia (TH) compared to TH was found in term gestation piglets, with reduced total cell death and increased oligodendrocyte survival but no improvement in aEEG recovery or magnetic resonance spectroscopy (MRS) at 48 h.^64^ By contrast, in preterm fetal sheep (0.7 of gestation, 28–30 weeks equivalent), an infusion of MgSO_4_ before acute HI somewhat improved white and gray matter gliosis and myelin density but did not improve long-term (3 weeks) EEG maturation or neuronal or oligodendrocyte survival.^65^

## Postnatal management

### Therapeutic hypothermia for preterm hypoxia-ischemia

The etiology of preterm brain injury is unequivocally multifactorial^13^, and in most cases, disability is not simply related to acute HI. Nevertheless, the risk of acute HI in preterm infants is greater than at term and highly associated with adverse outcomes.^1^ The use of therapeutic hypothermia (TH) in term infants has been proven in RCTs and meta-analyses to be safe and effective, improving disability-free survival at 18–24 months of age and reducing the risk of death or CP or IQ score <55 in 6- to 7-year-old children in high-income countries.^66–69^. In preterm infants, there is concern that TH might exacerbate complications of prematurity, including postnatal hypotension, intracranial bleeding, and respiratory compromise.^70^ In a cohort of neonates cooled outside standard TH criteria (*n* = 36, e.g., infants at 34–35 weeks gestation, with postnatal collapse or cardiac disease), compared with infants cooled as per protocol (*n* = 129), complication rates and neurologic outcomes at 18–20 months were similar; five neonates developed intracranial hemorrhage and had poor outcomes.^71^ In a retrospective cohort of 31 preterm infants born at 34–35 weeks gestation, TH appeared to be associated with increased mortality (12.9% vs. 0%, *p* = 0.04) and increased white matter damage (66.7% vs. 25.0%, *p* = 0.001), compared to 32 term infants.^72^ By contrast, a retrospective cohort study of preterm neonates (gestational age range 33–35 weeks) with HIE who underwent TH found similar rates of death or moderate-severe neurodevelopmental impairment in 11/22 cases^73^ compared to those in treated term infants in the large controlled TH trials.^67^

Preclinical evidence supports similar benefits and constraints in preterm animals. In preterm-equivalent fetal sheep, cerebral cooling for 72 h (with extradural temperature titrated to 29.5 ± 2.6 °C) started 90 min after severe asphyxia was associated with basal ganglia and hippocampal neuroprotection, protection of immature oligodendrocytes in periventricular and parasagittal white matter, and reduced overall microgliosis and apoptosis.^74,75^ Similarly, mild systemic hypothermia (37.2 ± 0.3 °C vs 39.5 ± 0.1 °C in normothermic controls) started 30 min after severe asphyxia, and continued until 72 h, reduced neuronal loss and microglial induction in the striatum, with faster recovery of spectral edge frequency, reduced seizure burden, and less suppression of EEG amplitude (*p* < 0.05).^76^ However, no benefit was seen when TH was commenced 5 h after HI.

Many questions remain to be answered for preterm neonates. It is plausible that TH may alleviate brain damage after acute perinatal asphyxia but not after chronic insults such as inflammation. Thus, the high rates of prenatal and postnatal infection/inflammation in preterm neonates might impair responses to TH.^77^ In postnatal day 7 rats exposed to HI, hemispheric and hippocampal protection with systemic cooling was lost after pre-insult sensitization with gram-negative lipopolysaccharide, but not a gram-positive mimic, PAM3CSK4,^78^ suggesting a pathogen-dependent effect. It is unknown whether these studies are relevant to the typical long-standing mixed effects of chorioamnionitis and postnatal inflammation. Combination therapies are promising but are largely unproven. Finally, the ideal protocol for identifying at-risk premature infants with acute neonatal encephalopathy remains unclear.

One multi-center randomized controlled trial is currently in progress testing the safety, feasibility and efficacy of TH within 6 h of birth in preterm infants at 33–35 weeks gestation with moderate to severe HIE (ClinicalTrials: NCT01793129). Publication of final results are awaited. Targeted use of TH for extremely preterm infants seems unlikely given the multiple morbidities (IVH, NEC, late-onset sepsis, BPD, apnea) during a prolonged hospitalization, which increases the risk of death or disability at 2 years of age. Moderate and late preterm infants are more likely to have identifiable, timed insults and may allow a focused use of TH similar to infants ≥36 weeks gestation at birth.

### Pluripotential (stem) cells

There is now considerable evidence from modern cohorts that neurodevelopmental disability after preterm birth is commonly associated with diffuse WMI, reflecting a persistently impaired white matter maturation.^16^ Persistent (tertiary) loss of trophic support and inflammation are key mechanisms.^20,30^ Exogenous pluripotential cells (stem cells) may promote angiogenesis, neurogenesis, synaptogenesis and neurite outgrowth, and so may have the potential to improve outcomes after preterm brain injury. Although its use to date has been primarily preclinical, a meta-analysis of neonatal rodent studies suggested that overall neural stem cells significantly reduced brain infarct size and improved motor and cognitive function.^79^ Many questions remain to be resolved, including the optimal dose, type of stem cells, and window of opportunity to start treatment.

Some conclusions can be drawn from the preclinical literature. Critically, it is very likely that multiple doses will be needed to see benefits. In term-equivalent, postnatal day 10 (P10) neonatal rats, for example, a single dose of umbilical cord blood (UCB) cells at P11 did not improve behavioral or neuropathological outcomes, whereas 3 doses (at P11, 13, 20) of UCB cells improved brain weight and behavioral deficits.^80^ Similar findings were seen in another term-equivalent study in P9 rats.^81^ In very preterm-equivalent rats, who underwent HI on postnatal day 3 and received 4 doses of human UCB cells over the next 3 days, UCBs were associated with improved behavioral outcomes at 27 days post HI.^82^ Consistent with this, in preterm fetal sheep, a single infusion of human amnion epithelial cells (hAECs) up to 24 h after HI was anti-inflammatory, anti-gliotic, and neuroprotective in the hippocampus, but had limited effect on numbers of oligodendrocytes or the proportion of immature/mature numbers of oligodendrocytes after 7 days recovery and had no effect on recovery of EEG activity.^83^ By contrast, in the same paradigm, repeated and delayed intranasal infusion of hAECs at 1, 3 and 10 days after umbilical cord occlusion for 25 min was associated with improved brain weight and restoration of immature/mature oligodendrocytes and fractional area of myelin basic protein, with reduced microglia and astrogliosis. Neuronal survival in deep gray matter nuclei was improved, with reduced microglia, astrogliosis and cleaved-caspase-3-positive apoptosis. Functionally, cortical EEG frequency distribution was partially improved.^84^

Further, hAECs may alleviate inflammatory injury. In preterm fetal sheep, after lipopolysaccharide (LPS) infusions for 3 consecutive days at 109, 110 and 111 days of gestation, 3 intravenous doses of 60 million hAECs given starting at 110 days were associated with attenuation of activated microglia, reduced number of pyknotic cells and significantly increased number of oligodendrocytes and myelin basic protein compared to animals treated with LPS only.^85^ Thus, overall, there is good large animal evidence supporting the benefit of a reasonably wide window of opportunity with intermittent infusions of stem cells, although more detailed studies are needed to establish the ideal protocol.

There have been several rather heterogeneous Phase I/II clinical trials in preterm infants as recently reviewed.^86^ Many were focused on treating chronic deficits rather than perinatal injury. Overall, these studies support the general safety of stem cells and that there may be potential benefits, but definitive studies are still lacking. A safety and feasibility study in infants under 28 weeks gestation, using autologous UCB mononuclear cells via intravenous injection at a dose of 25–50 million cells/kg in 20 infants, is underway.^87^

### Anti-inflammatory interventions

There is growing evidence that there is a tertiary phase of ongoing inflammation and apoptosis for weeks, months or even years after perinatal injury and that this likely contributes to diffuse injury and dysmaturation of the developing brain.^20^ For example, in postmortem studies, premature infants exposed to HI can have stalled maturation of oligodendrocytes and chronic inflammation^16,88^ and impaired connectivity on MRI studies.^89–91^ Notably, many clinical cases of cystic WMI are not seen until several weeks after birth.^31,32^ This delayed damage could, in principle, reflect the slow evolution of injury or multiple injurious inputs over time. Both are likely contributing, as suggested by a cohort study that found that combinations of HI, infection and inflammation could all ultimately be associated with injury.^92^

Preclinical studies strongly support the concept that cystic WMI can develop in the tertiary phase even after a single episode of HI. For example, in P7 neonatal rats, necrotic brain lesions were found to evolve from 24 h to 21 days after.^93^ Moderate HI was associated with the slowest evolution of brain necrosis. Similarly, in preterm fetal sheep, at 3 and 7 days after global HI induced by complete occlusion of the umbilical cord for 25 min, HI led to diffuse WMI injury, with selective loss of mature oligodendrocytes, increased microglia and impaired myelination, like the typical pattern of diffuse WMI in most recent human postmortem studies.^28,94^ However, unexpectedly, 14 to 21 days after HI, cystic lesions developed in the temporal white matter.^30^ Intriguingly, the regional distribution of this cystic white matter degeneration at 21 days after HI was closely related to the location of dense microglial aggregates visualized at earlier time points, raising the possibility that exuberant inflammation may contribute to late necrosis. Strongly supporting this concept, in a subsequent study, intracerebroventricular infusion of the soluble tumor necrosis factor (TNF) inhibitor, etanercept, at 3, 8 and 13 days after umbilical cord occlusion attenuated cystic WMI and restored oligodendrocyte maturation and deficits in myelin protein expression.^95^ These encouraging results suggest that, although far more knowledge is needed, delayed anti-inflammatory interventions may be a promising strategy to reduce neurodevelopmental disabilities. Erythropoietin, azithromycin, melatonin, and caffeine citrate have been the subject of extensive preclinical and clinical studies and will be discussed in detail. Other repurposed anti-inflammatory medications are also under investigation, both preclinically and clinically. This includes anakinra, an interleukin 1 receptor antagonist, which is the subject of a safety and feasibility study in preterm infants between 24 and 28 weeks gestation, that aims to decrease the morbidity and mortality of prematurity attributed to inflammation, including bronchopulmonary dysplasia, gut injury and necrotizing enterocolitis, and brain injury.^96^

#### Erythropoietin

Erythropoietin (Epo) is an endogenous growth factor whose primary function is to stimulate erythropoiesis. However, Epo receptors (EpoR) are also present on neuron progenitor cells,^97^ neurons,^98^ astrocytes,^99^ oligodendrocytes,^100^ microglia,^101^ and endothelial cells.^97^ Epo-bound receptors dimerize to activate anti-apoptotic pathways via phosphorylation of JAK2, phosphorylation and activation of MAPK, ERK1/2, as well as the PI3K/Akt pathway and STAT5, which are critical in cell survival.^101,102^ Epo also has indirect effects, increasing iron utilization by increasing erythropoiesis and by decreasing inflammation^103,104^ and oxidative injury.^105,106^

In preclinical models of neonatal brain injury, recombinant human Epo has anti-inflammatory,^103,104,107–111^ anti-excitotoxic,^112^ antioxidant,^113^ and anti-apoptotic effects on neurons and oligodendrocytes, and promotes neurogenesis and angiogenesis,^114^ which are essential for injury repair and normal neurodevelopment. Epo effects are dose-dependent, and repeated doses are more effective than single doses^115–117^ at reducing learning impairments following brain injury.^118,119^ Encouragingly, in P7 rats, although starting Epo 48 h after HI did not improve the area of infarction, it improved behavioral outcomes, with enhanced neurogenesis, increased axonal sprouting, and reduced WMI.^120^ However, this should be interpreted with caution. In preterm fetal sheep, a high dose infusion of Epo from 30 min after asphyxia until 72 h was associated with partial neuroprotection, with improved electrophysiological and cerebrovascular recovery after 1 week of recovery and reduced apoptosis and inflammation.^121^ When the infusion was delayed until 6 h after asphyxia, there was no histological or electrophysiological protection.^122^ Of concern, in the same paradigm, a clinical regime of high-dose intermittent boluses of Epo started at 6 h was associated with impaired EEG recovery and bilateral cystic injury of temporal lobe intragyral white matter in the majority of fetuses,^122^ suggesting that unless Epo can be started immediately, it is unlikely to be beneficial for preterm HI brain injury.

Clinical evidence supports this concern. Five clinical trials of Epo for neuroprotection of infants <37 weeks have now been published,^123–127^ four of which were reviewed in a meta-analysis by Fischer et al., suggesting that prophylactic Epo improved the cognitive development of preterm infants.^128^ However, significant differences in the patient populations (mean gestational age 25.9 to 30.4), Epo dose (400 U/kg to 3000 U/kg) and duration of therapy (3 days to 12 weeks) make it difficult to reach definitive conclusions. Critically, the two largest trials, the Swiss trial^125^ and the Preterm Epo Neuroprotection Trial (PENUT)^127^ did not show any improvement in the combined outcome of death or severe neurodevelopmental impairments at 2 years of age. Epo also did not affect inflammatory markers in the PENUT trial, despite data in rodents that this was one of the mechanisms of action.^129^ Potential reasons why Epo was ineffective include important differences between preterm infants and preclinical models, incorrect timing of the intervention, incorrect dosing schedule or the possibility that iron deficiency in the Epo-treated group contributed to the lack of benefit in children enrolled in the PENUT Trial.

#### Melatonin

Melatonin (N-acetyl-5-methoxytryptamine) is a naturally occurring indolamine secreted by the pineal gland to regulate circadian rhythm.^130^ Melatonin is safe in pregnancy and in neonates, readily crosses the placenta and blood-brain barrier, and is a potent antioxidant with potential for neuroprotection after inflammation and HI in high-risk preterm fetuses and infants.^130^

In preterm fetal sheep at 0.7 gestation (98–99 days of gestation, term gestation being 147 days), a maternal infusion of low-dose melatonin dissolved in ethanol from 15 min before umbilical cord occlusion until 6 h after was associated with faster recovery of EEG activity, delayed onset of seizures, improved survival of mature oligodendrocytes, and reduced microglial activation in the periventricular white matter.^131^ The ethanol vehicle was independently associated with reduced duration of fetal seizures and improved neuronal survival in the striatum, but worse neuronal survival in the hippocampus and less white matter proliferation compared to saline treatment. Similarly, fetal infusion of melatonin dissolved in ethanol over 24 h in preterm fetal sheep, starting 2 h after HI, was associated with region-specific improvements in white matter damage 10 days after HI.^132^ Similarly, independent and combined neuroprotective effects of melatonin and ethanol were confirmed in term neonatal piglets.^133,134^ The EMA and FDA have recognized that ethanol is an active or adjuvant excipient for melatonin and is likely to be safe at levels below that recommended by the FDA. The American Academy of Pediatrics Committee on Drugs recommended that the amount of ethanol in any preparation should not lead to blood concentration >0.25 g/L after a single dose in term infants.^135^ A multicentre international phase I dose escalation study of melatonin as an adjunct with TH (ACUMEN Study—Acute high dose Melatonin for Encephalopathy of the Newborn) is starting using a melatonin/ethanol formulation in term infants. As the oral bioavailability of melatonin is around 15%, intravenous administration is necessary.^136^

Small clinical studies suggest that, in preterm infants, oral melatonin is safe and may improve survival in septic shock and reduce lung injury associated with ventilation.^137,138^ Further, in a randomized double-blind placebo-controlled pilot study, oral melatonin given once a day was associated with lower levels of F2-isoprostanes at 48 h.^139^ This result may be significant given the association of increased levels of F2-isoprostanes with abnormal white matter injury scores at term-corrected age.^140^ Long-term follow-up has not been reported and is essential.

A recent review discusses the preclinical evidence for melatonin as a therapeutic agent for preterm brain injury and calls for well-designed preclinical research to understand the dosing and timing of administration and neonates who might benefit from melatonin.^141^ Nevertheless, there is intense interest in the entrainment of the circadian rhythm in high-risk neonates in the NICU and its effect on later cognitive development; this is being studied in the “Circa Diem” Study.^142^

#### Caffeine citrate

Caffeine citrate, a methylxanthine, is routinely used in preterm infants with the primary intention of preventing apnea of prematurity (AOP) and in an attempt to decrease the risk of unsuccessful extubation attempts. These indications are based on the results of the CAP (Caffeine for Apnea of Prematurity),^143^ other caffeine RCTs and resulting Cochrane meta-analyses.^144,145^ The CAP trial randomized 2006 infants less than 1250 g to caffeine citrate or placebo and performed rigorous follow-up of recruited infants. Caffeine citrate for AOP decreased bronchopulmonary dysplasia and time requiring respiratory support with no significant side effects. Caffeine given for AOP reduced the incidence of CP (4.4% vs. 7.3%; adjusted odds ratio, 0.58; 95% CI, 0.39 to 0.87; *p* = 0.009) and of cognitive delay (33.8% vs. 38.3%; adjusted odds ratio, 0.81; 95% CI, 0.66 to 0.99; *p* = 0.04) at 18 to 21 months.^146^ The significant improvement in disability-free survival was not seen when infants were followed at 5 years of age.^147^ At 11 years of age, neurobehavioral outcomes were similar between groups, but the caffeine-treated group performed better in fine motor coordination (mean difference (MD) = 2.9; *p* = 0.01), visuomotor integration (MD = 1.8; *p* < 0.05), visual perception (MD = 2.0; *p* = 0.02) and visuospatial organization (MD = 1.2; *p* = 0.003).^148^ The caffeine-treated group also had a reduced risk of motor impairment.^149^ A number of mechanisms underlie the potential neuroprotective effects of caffeine citrate in premature infants. Caffeine is an adenosine receptor antagonist and, by this mechanism, increases respiratory neural output and also enhances CO_2_ responsiveness.^150^ Preclinical studies testing caffeine after HI in a neonatal premature model demonstrated improved function, reduced brain injury, decreased apoptosis, anti-inflammatory actions, increase in myelination and promotion of oligodendroglial in treated animals.^151–154^

### Seizure management

Preterm neonates have high rates of clinical and subclinical seizures.^155,156^ Neonatal seizures are associated with poor neurodevelopment, and this may be even more severe in preterm than term infants.^157^ In experimental studies, there is often little relationship between seizure control and neurohistological outcomes.^158^ For example, in preterm fetal sheep, intravenous infusion of MgSO_4_ for 24 h before asphyxia until 24 h after asphyxia was associated with a marked reduction of the post-asphyxial seizure burden, confirming its physiological anti-excitotoxic role but did not improve neuronal survival and indeed exacerbated loss of oligodendrocytes after 7 days.^159^

Of further concern, in animal studies, many anticonvulsant drugs are neurotoxic at clinical and subclinical levels.^160^ For example, in P7 rats, when brains are equivalent to the late preterm infant, clinically relevant doses of phenytoin, phenobarbital, diazepam, clonazepam, vigabatrin and valproate were associated with widespread apoptotic degeneration, reduced expression of pro-survival neurotrophins, and a significant reduction in brain weight after eight days. Further, in P7 rats, clinical doses of phenobarbitone, phenytoin and lamotrigine, but not levetiracetam, were associated with impaired striatal synaptic development between P10 and P18.^161^ The optimal management of seizures in preterm neonates is a high priority for research.

### Non-pharmacological neuroprotection

Beyond medications that can be prescribed or novel procedures that can be performed, the care practices that are provided daily have an impact on both the prevention of acquired brain injury and the promotion of healthy brain maturation. Models of care that reduce separation, promote family integration into care, and focus on creating a neuro-supportive environment are rooted in evidence and, at the same time, require further study to be able to optimize outcomes for preterm infants and their family units.^162^

Specifically, immediate skin-to-skin care, otherwise known as kangaroo care, has been shown to reduce preterm mortality globally,^163^ and has been linked with improved biomarkers of resilience and stress reduction in both mothers and infants. Skin-to-skin care has also shown benefits for microbiome development and stabilization of respiratory physiology, both of which may also influence neurodevelopment.^164,165^ Early and frequent skin-to-skin care for preterm infants results in decreased length of exposure to mechanical ventilation and oxygen and overall decreased length of stay for the initial neonatal hospitalization.^166^

Nutrition is critical to brain development. Promotion of maternal breast milk or donor breast milk if maternal breast milk is unavailable, early enteral nutrition and early fortification have been linked to reduction of growth failure and improved brain health outcomes and should be an important focus on neuroprotective care.^167,168^

Evidence supports that both pain and our treatment of pain impact brain development and neurodevelopmental outcomes in preterm infants. Both prophylactic and procedural opioid exposure specifically has been linked to cerebellar injury, decreased cerebellar volume, and poorer neurodevelopmental outcomes in early childhood.^169–171^ Treating pain non-pharmacologically as well as reducing pain have shown to be neuroprotective. Efforts should be made to minimize unnecessary procedures, particularly skin breaks, in premature neonates, as these have been linked to altered thalamic and microstructural development and have an independent impact on childhood cognitive outcomes.^172–175^ Recognizing that all pain is not avoidable in our efforts to support the health and development of preterm children, many neuro-supportive care practices can also be used to minimize the impact of pain, including maternal breast milk and skin-to-skin care.^176^ Pharmacological agents such as sucrose can be effective in reducing the pain experienced by neonates undergoing mildly painful procedures.^177^ Sucrose should be used judiciously as the underlying mechanism is still unknown, and the long-term effects on development and potential for hyperalgesia are unknown.^172^ The preterm infant spends most of its time asleep, and unlike term and young infants, the majority of time is spent in active sleep (AS). The ultradian cycling between AS and quiet sleep (QS) is thought to be important in brain development and interrupted sleep is associated with adverse neurodevelopmental outcomes in both preterm human infants and experimental animal models.^178^ In addition, improving the timing of hands-on care or interventions in order to safeguard sleep has also been linked to improved brain health and development.^179^

Models of care, such as family-centered care (FCC)^180^ or family-integrated care (FiCARE),^181^ both of which are centered around the concept of Families as Partners (VON),^182^ are linked to intermediate outcomes of better family engagement, which can in turn lead to improved neurodevelopment.

Together, these care practices have an opportunity to optimize brain development and are within the control of neonatal intensive care providers and units to a growing body of literature demonstrating the feasibility of implementation.^183^

### Neuroprotection and neuroplasticity bundles

Preterm brain injury is a complex and multifactorial process.^184^ Genetic, epigenetic, metabolic, prenatal, perinatal and postnatal factors interact to protect, cause or exaggerate neonatal brain injury.^185–188^ Given the complexity of the issue, there is an increasing interest in a “bundled” approach using quality improvement methodology to prevent acute preterm brain injury.^189^ Neuroprotection bundles can be divided into (1) acute brain injury prevention and (2) neuroplasticity bundles.

The key interventions in the preterm acute brain injury prevention bundles are early identification of high-risk pregnancies and in-utero transfer,^190^ prevention of fluctuation in physiologic parameters such as partial pressure of carbon dioxide, blood pressure, and temperature at birth,^191–193^ prevention of acidosis,^194^ midline head position,^195^ minimizing painful procedures,^196^ and maintenance of electrolyte balance (especially serum sodium).^197^ Recent studies showed the implementation of such neuroprotection bundles targeting some or all those key elements using Quality Improvement (QI) methodology decreased the incidence of acute brain injury and improved long-term neurodevelopment outcomes in extremely premature infants born at and outside tertiary centers.^198–202^

Neuroplasticity interventions, unlike those for acute brain injury, target potential brain injury and growth well beyond the first few days of birth and after discharge.^203^ The key elements of such bundles include the non-pharmacological interventions discussed in detail above, including empowering families through family-centered care model;^181^ optimizing nutrition;^204^ developmental care;^205^ skin-to-skin care and massage therapy;^206,207^ positive stimulating sounds such as music therapy and reading programs,^208^ parental voice,^209,210^ minimizing disturbing noises,^211^ enhancing physiologic sleep-wake cycles^212^ and encouraging positive social interaction.^213^

**Fig. 3:**
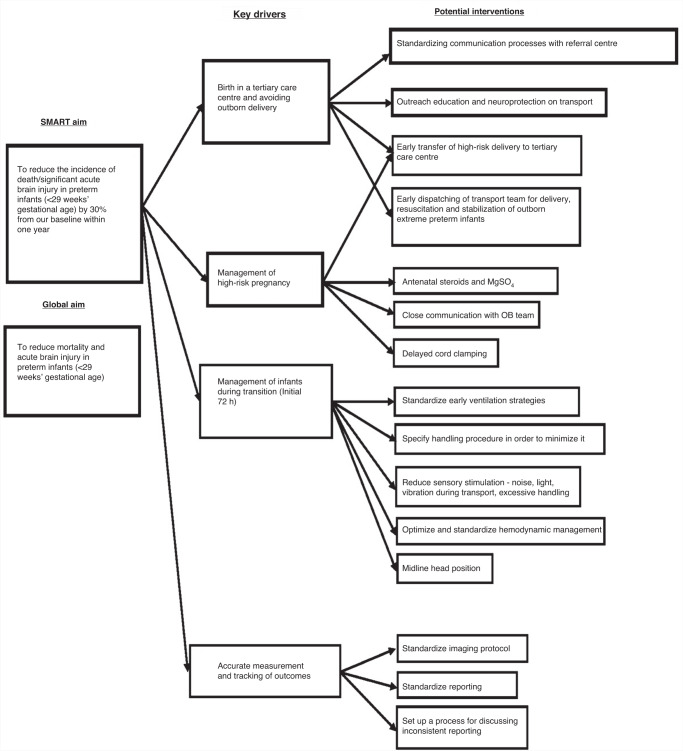
Example of using QI methodology, a driver diagram and smart aim to reduce IVH.

Compared to acute brain injury prevention bundles, there is emerging evidence that parental involvement^214^ and neuroplasticity interventions may improve long-term cognitive and motor outcomes.^206–208^ These studies were relatively small, and studies with a larger sample size are now needed to confirm these encouraging preliminary findings. Figure 3 shows an example of using QI methodology for premature infants, with a driver diagram and smart aims to reduce IVH.

### Social determinants of neurodevelopmental outcomes

Evidence from multiple cohort studies and the resultant meta-analyses have shown that the impact of a number of social determinants has an impact on the neurodevelopmental outcomes of infants and that this risk is magnified in infants who are born preterm.^7,215–217^ A prospective cohort study of 226 infants born between 26 and 32 weeks gestation examined the impact of socio-economic status (SES) as a moderator of outcome, showed that SES had the same statistical association with cognitive outcome as WMI and that SES moderated the long-term outcomes of infants with brain injury and chronic lung disease.^218^ An MRI study of preterm infants, excluding those with severe brain injuries, showed regional differences in brain morphology associated both with premature birth and SES and that these factors interacted. Lower maternal education as a proxy for SES has been widely studied in this context, perceptions of neighborhood safety and the maternal vulnerability index, a composite measure that reflects physical, social, and health care needs, have also been associated with preterm birth, less use of prenatal care and poorer long-term outcomes.^219,220^ While the mechanisms underlying these associations are not fully elucidated, these findings suggest that strategies improving social determinants of health for premature infants may have a positive effect on their long-term health and neurodevelopmental outcomes^221^ and that, as such, advocacy by clinicians in this area is vital.

## Conclusion

There has been little change in the rather high burden of lifelong disabilities after preterm birth in recent years, despite continuing improvement in survival. Developing therapeutic strategies for preterm brain injury is challenging because of the heterogeneous nature of preterm brain injury, including exposure to antenatal and postnatal hypoxia, acute perinatal asphyxia, prenatal and postnatal infection/inflammation, ventilation-induced brain injury and risk of side effects associated with treatments, including anticonvulsants and glucocorticoids. Each premature infant is likely exposed to different combinations of factors, As highlighted in this review, several potential neuroprotective interventions have shown promise in preclinical studies and/or in clinical trials. With further well-designed, translational research, these treatment strategies may help to reduce the high burden of brain damage and disability associated with preterm birth. Neurorestorative interventions targeting the repair of the injury should also be investigated. In the meantime, bundles of care protecting and nurturing the brain in the neonatal intensive care unit and beyond should be widely implemented in an effort to limit injury and promote neuroplasticity.

## Data Availability

Data sharing is not applicable to this article as no datasets were generated or analyzed during the current study.
